# Treatment of Multiple Primary Malignancies With PD-1 Inhibitor Camrelizumab: A Case Report and Brief Literature Review

**DOI:** 10.3389/fonc.2022.911961

**Published:** 2022-07-05

**Authors:** Yuchen Wan, Zhixue Wang, Ning Yang, Fenye Liu

**Affiliations:** ^1^ Department of Traditional Chinese Medicine, Shandong Provincial Hospital Affiliated to Shandong First Medical University, Jinan, China; ^2^ The First Faculty of Clinical Medicine, Shandong University of Traditional Chinese Medicine, Jinan, China; ^3^ Department of Radiation Oncology, Shandong Provincial Hospital Affiliated to Shandong First Medical University, Jinan, China

**Keywords:** multiple primary malignancies, immune checkpoint inhibitors, camrelizumab, immunotherapy, acquired resistance

## Abstract

**Background:**

With significant advances in the diagnostic tools and treatment modalities of cancer, the incidence of multiple primary malignancies (MPMs) has increased in the last decades. The therapeutic option changed with the arising of immune checkpoint inhibitors (ICIs), which have improved the survival of a broad spectrum of tumors. However, little information is available when it comes to the efficacy, resistance, and underlying mechanisms of ICIs.

**Case Presentation:**

A 67-year-old woman was diagnosed with pulmonary sarcomatoid carcinoma (PSC) with a history of hepatocellular carcinoma (HCC) and viral hepatitis B. Following the lack of response to systemic chemotherapy, she was treated with camrelizumab, an anti-programmed cell death protein 1 monoclonal antibody, in combination with chemotherapy, and a partial response was obtained both in PSC and HCC. After a course of 9-month treatment, the PSC lesion shrank still, while HCC was evaluated as a progressive disease with an increase in the diameter of liver neoplasm, elevated alpha-fetoprotein, and enlarged abdominal lymph nodes. Then, with the addition of radiotherapy for abdominal metastasis, the lung lesion was continuously shrinking. In the meantime, the liver neoplasm and abdominal lymph nodes showed no significant enlargement.

**Conclusion:**

Camrelizumab combination therapy could consistently benefit the MPM patients with PSC and HCC, which may be a promising option for patients with MPMs.

## Introduction

Multiple primary malignancies (MPMs) were firstly described by Billroth and defined as the concurrence of two or more different neoplasms in the same individual. Each cancer originates from a single primary site and is neither an extension, recurrence, or metastasis (1). With the evolution in the diagnosis and treatment of cancer, a higher proportion of surviving patients with cancer is at a greater risk of developing a second, third, or even a higher number of primary malignancies. The frequency of multiple primaries ranges between 2 and 17% (2). Moreover, the incidence of MPMs was 13% among 24,859 patients registered with incident cancer from the Swiss cancer registries (3). Aging and cancer treatment, such as conventional radiation therapy and chemotherapy, may contribute to the development of MPMs (4). Pulmonary sarcomatoid carcinoma (PSC) is a rare subtype of non-small cell lung cancer characterized by epithelial-to-mesenchymal transition, highly aggressive peculiarity, and propensity for metastasis (5). Its prognosis is poor, the patients with PSC have a median survival of less than 12 months (6), and the 5-year survival rate is limited to 15–25% (7). Hepatocellular carcinoma (HCC), as the most common type of primary liver cancer, is ranked as the sixth most common neoplasm and the third leading cause of cancer-related mortality worldwide in 2020 (8). The improvement in early cancer diagnosis and comprehensive treatment leads to the prolonged survival of cancer individuals, and the number of MPM patients with HCC successively increases (4). It was reported that lung cancer and colorectal and thyroid carcinoma usually complicate with HCC (9). PSC is a rare extra-hepatic tumor due to its low incidence.

MPM is a major medical problem which is common in clinical practice. Due to the difference in pathological properties, the sensitivity to drug therapy varies between different molecular phenotypes. How to specify and optimize the treatment strategies of MPMs has become a thorny problem perplexing the doctors at present. Over the past decades, immunotherapy has sprung up and becomes the cornerstone in the progress of treatment of MPMs. Immune checkpoint inhibitors (ICIs) are the most important and well-studied immunotherapeutic agents with rapid breakthroughs in the extensive application of tumor therapy. Due to the long and durable responses and good tolerance, ICIs have been widely used for the treatment of melanoma, non-small cell lung cancer, esophageal cancer, and other solid tumors and hematologic malignancies not only in the metastatic but also in the adjuvant settings (10–12). ICIs work by blocking the combination of checkpoint molecules, such as cytotoxic T lymphocyte-associated protein 4, programmed cell death protein 1 (PD-1), and programmed death ligand 1 (PD-L1), thereby resulting in the robust activation of the immune system and productive antitumor immune response (13). Due to a high rate of chemoresistance to platinum-based standard regimens and low response to radiotherapy, the current treatment options for PSC is limited (14). ICIs may potentially reform the remedial scenery and clinical outcome of PSC given that PSC appears to be a hot tumor with high immune and inflammatory cell infiltration and that PD-L1 is also highly expressed in PSC tissue (53–70%) (5, 15, 16). In advanced HCC, ICIs have shown clinically relevant benefits as monotherapy or in combination with TKI/antiangiogenetic agents and local treatment (17–20). Importantly, camrelizumab, an anti-PD-1 inhibitor, has been approved for the second-line treatment of advanced HCC patients by the National Medical Products Administration of China and granted the Orphan Drug Designation in the treatment of HCC by the United States Food and Drug Administration (21).

Because of the better data on immunotherapy for multi-tumor species, ICIs may offer a promising avenue for MPMs. Nonetheless, limited clinical evidence is available for their efficacy. Despite the fact that many patients experience dramatic tumor regression in response to ICIs, most patients and cancers did not respond to these therapies due to primary resistance and acquired resistance, which have overlapping tumor intrinsic and micro-environmental extrinsic factors responsible for the ICI resistance (22). Herein we report a case of an MPM patient with PSC and HCC who benefit from camrelizumab combination therapy.

## CASE PRESENTATION

A 67-year-old female complained of a 1-week history of one right lung mass and was admitted to Shandong Provincial Hospital Affiliated to Shandong First Medical University in July, 2020. She presented with shortness of breath, dry cough, and chest tightness, without hemoptysis or chest pain. The patient suffered from stage IA (cT1aN0M0) HCC, which was diagnosed in February, 2017 in Qi Lu Hospital. The prior liver magnetic resonance imaging result revealed an abnormal signal (arterial phase hyperenhancement and delayed phase hypointensity) in the anterosuperior segment of the right liver lobe. Then, she underwent the transcatheter arterial chemoembolization (TACE) treatment on HCC. The patient had a 7-year history of hepatitis B virus (HBV) with antiviral drug therapy; no family history was described.

**Figure 1:**
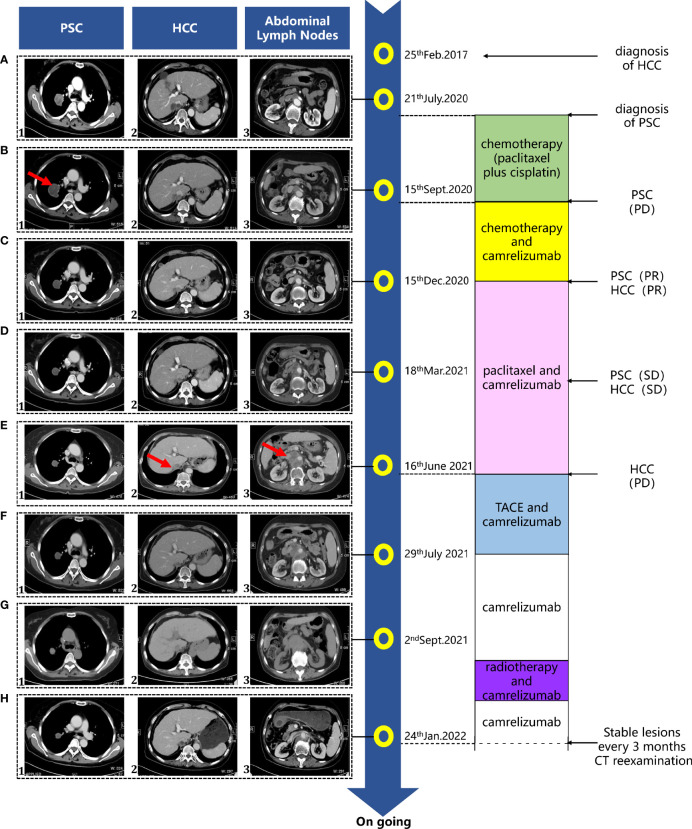
Treatment timeline of the patient. Progressive lesions are indicated by red arrows. PD, progressive disease; PR, partial response; SD, stable disease.

After admission to the hospital, the laboratory examination revealed that the liver function test was within normal range. The viral markers showed positive serum hepatitis B surface antigen, hepatitis B e antigen, and hepatitis B core antibody. The oncological marker alpha-fetoprotein (AFP) was 5.1 ng/ml, and carcinoembryonic antigen was 2.0 ng/ml. The workup was pursued by a computed tomography (CT) scan, which showed a right lung mass (3.6 cm × 3.7 cm), a hepatic lump (2.2 cm × 1.5 cm) next to the inferior vena cava, and enlarged retroperitoneal lymph nodes (**Figure 1**). The primary clinical stage of the lung tumor was identified as IB (cT2N0M0). Furthermore, she was diagnosed with sarcomatoid carcinoma as the histopathologic results from the pulmonary mass biopsy showed the following: CK (AE1/AE3) (weakly +), TTFI (–), Glypican-3 (NS), CK8/18 (+), P40 (–), Syn (–), CD20 (–), LCA (–), CD3 (–), CD30 (–), Ki-67+ (20%), SMA (–), and vimentin (+).

Considering her good clinical conditions, the patient was treated by chemotherapy with paclitaxel (150 mg/m^2^) plus cisplatin (75 mg/m^2^) every 21 days for two cycles. Unfortunately, on September 15, 2020, a whole-body CT scan showed a progressive disease with an increase in the diameter of the right lung lesion (4.6 cm × 4.2 cm) (**Figure 1**).

**Figure 2:**
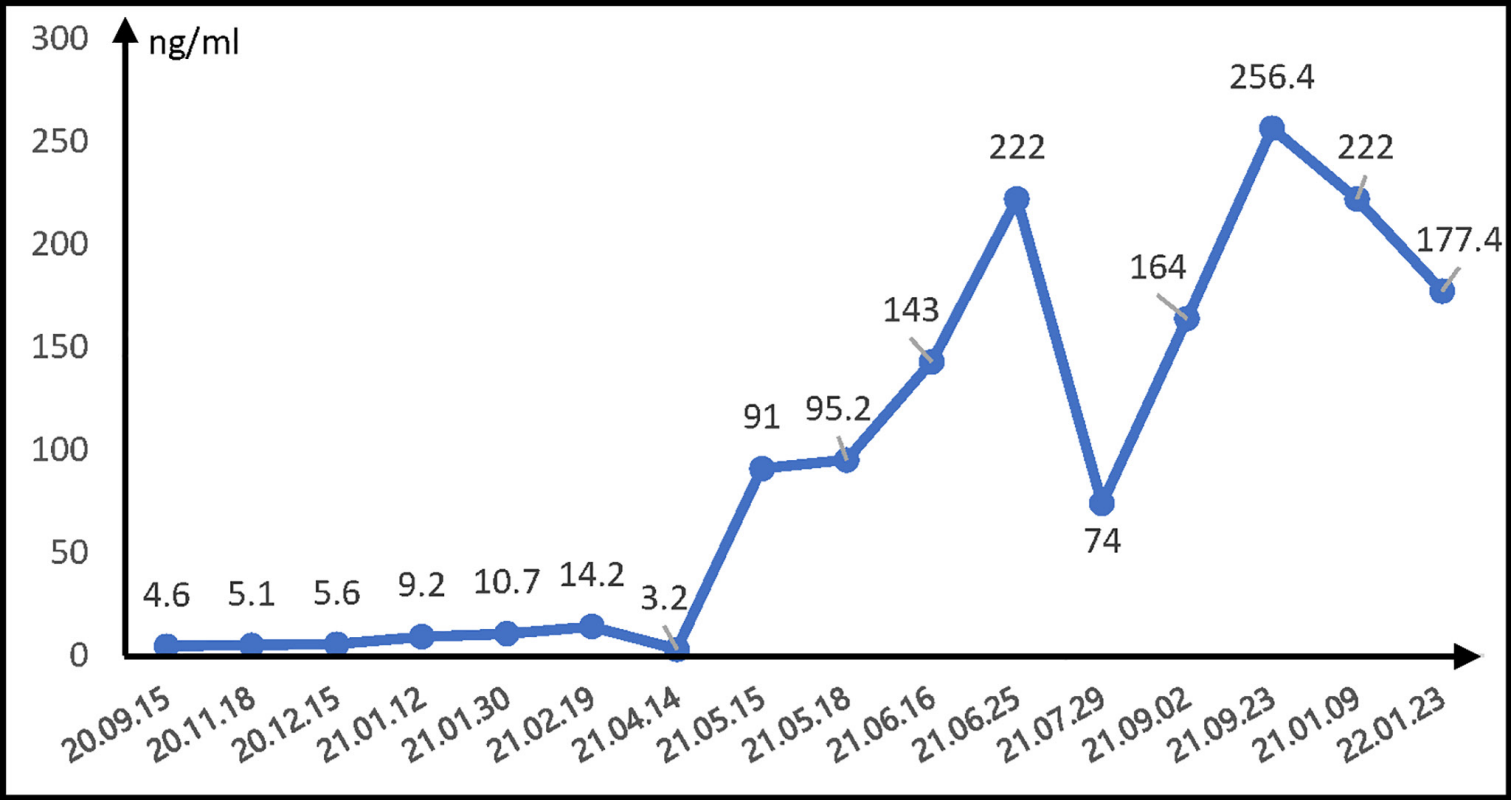
Changes of serum AFP level during camrelizumab treatment. AFP, α-fetoprotein.

Thus, the patient received comprehensive genomic profiling by next-generation sequencing in lung puncture tissue from the previous biopsy. The immunohistochemical results showed the expression of PD-L1 as 45% and the tumor proportional score as >1%. All things considered, on September 24, 2020, the anti-PD-1 mAb camrelizumab (200 mg, every 21 days) was added to the foregoing chemotherapy with cycles of every 21 days. After 3 cycles of camrelizumab, the patient was evaluated as with partial response for both PSC (3.1 cm × 3.1 cm) and HCC (1.4 cm × 1.2 cm) on the basis of CT imaging on December 15, 2020 (**Figure 1**). Then, the combination therapy with camrelizumab and paclitaxel was adopted as maintenance treatment. A CT scan was conducted approximately every 3 months, while a laboratory test was performed prior to every treatment. Both PSC and HCC were assessed as stable disease (SD) (**Figure 1**). Unfortunately, on June 16, 2021, the patient was assessed as PD with an increase of live tumor (the longer diameter is 1.5 cm) associated with the rise of AFP (**Figure 2**) and the significant enlargement of abdominal lymph nodes in a whole-body CT scan (**Figure 1**). The patient underwent the TACE treatment while remaining off from paclitaxel on the account of third-degree myelosuppression. Camrelizumab was still carried on, and the PSC lesion continued to shrink. On September 2, 2021, to figure out the metastasis of the abdominal lymph nodes, local radiotherapy (2 Gy each time for 25 times; in total, 50 Gy) was added to the camrelizumab treatment. After 23 cycles of camrelizumab treatment, the efficacy was assessed as SD for PSC and HCC according to the CT scan image in the last follow-up (**Figure 1**).

In the course of the camrelizumab combination therapy, no treatment-related toxicities were observed. Throughout the process, camrelizumab showed a manageable safety profile and acceptable tolerability, with no treatment-related serious adverse events.

## Discussion

Cancer survivors have an increased risk of developing MPMs despite early detection and improved treatment regimens. MPMs are known to occur at a high incidence rate in the malignancies of head and neck (23), respiratory, gastrointestinal, and genitourinary systems (24). The patient harbored two primary malignancies, HCC, and secondary PSC. HCC is one of the most common malignancies worldwide. Consequently, it is common to find MPM patients with HCC over a long-term follow-up. In MPM patients with HCC, the most common extra-hepatic malignancies were lung cancer and colorectal and thyroid carcinoma. Given the low incidence of PSC, MPM patients involving PSC and HCC are even rarer.

The therapeutic options for MPMs are usually limited (25). To our knowledge, regarding ICI treatment for MPMs, there have been only a few case reports available and evidence-based studies are lacking. There are several immune checkpoint inhibitors, such as nivolumab, pembrolizumab, and atezolizumab, which have demonstrated encouraging efficacy in the treatment of multiple primary tumors (26–30). A bulk of studies have shown the favorable efficacy of ICIs on PSC (31–33) and HCC (34–36), respectively. Based on the promising outcome of ICIs in HCC, PSC, and MPM patients, this is the first attempt of camrelizumab used for the patient with PSC and HCC, though it has been demonstrated to have anti-tumor activity in HCC patients in the absence of evidence for PSC conventional effective therapies.

In this case, the PSC lesion showed partial remission and durable response to camrelizumab combination therapy after progression with systemic chemotherapy, while on the other hand, the HCC lesion showed a partial remission with a duration of 9 months and gradually progressed with enlarging focus of hepatocellular carcinoma, elevated AFP, and abdominal lymph nodes. Within the continuous treatment of camrelizumab for less than 2 years, the duration of the PSC lesion in maintaining partial remission was more than 17 months. A previous study demonstrated that a single-agent immunotherapy failed to lead to benefit survival for HCC (37). In this case, after HCC progressed, camrelizumab/TACE/radiotherapy combination was introduced sequentially. The abovementioned indicators of HCC were improved, and the patient was in good condition with acceptable safety.

The etiology of MPMs is categorized into three major groups: treatment-related neoplasms, syndromic cases, and common etiologic factors, such as genetic predisposition or some environmental factors (38). Immune escape is a common pathological mechanism of various malignant tumors (39), which may be one of the important mechanisms in the development of MPMs and make ICIs to be the underlying treatment options for various MPMs (2). ICIs work by activating previously primed T cells, which have lost effector and proliferative functions, to recover their antitumor effects. In addition to T cells, other innate and adaptive immune cells can be activated by ICIs to orchestrate an effective response against tumors (40). Furthermore, ICIs inhibit not only the PD-1/PD-L1 axis but also the PD-L1/CD80 cis-interactions on dendritic cells (DCs), freeing more CD80 molecules to promote T cell priming (41). Genomic instability is usually considered as a crucial factor in various carcinogenesis (42). In MPMs, as previously mentioned, the main etiologies can be classified as cancer treatment-related, co-exposure-related, or other factors. Exposure to chemotherapy might not directly cause baseline genomic instability, but it may increase the mutational profile of a tumor (43). We therefore speculate that, in addition to the anti-tumor effects of ICIs, MPMs have extensive genomic instability, which could form a large antigen pool and increase the chances for T cell recognition, resulting in a better ICI outcome (44).

In spite of promising results from clinical studies, only a small subset of patients respond to immunotherapy and benefit from ICI-based treatments due to the resistance of ICIs (45, 46). The underlying mechanism of ICI resistance involves multiple defects in key elements required for optimal T-cell response. Specifically, the absence of tumor antigens and defective antigen presentation are the main tumor-cell-intrinsic factors for resistance; at the same time, some immunosuppressive components in the tumor microenvironment, including regulatory T-cells (Tregs), myeloid-derived suppressor cells (MDSCs), and inhibitory immune checkpoints, are important extrinsic factors for immunotherapy resistance (47).

Liver itself develops a specific immune tolerance to reduce abnormal immune responses to large amounts of antigens. Besides this, chronic HBV infection is associated with the peripheral tolerance and tumor immunosuppressive microenvironment (48, 49). The potential mechanisms of immune resistance in HCC can be attributed to the following four ways (1): Under the pressure of immune selection, HCC escaped immune surveillance through losing human leukocyte antigen heterozygosity and evolving into a more immunosuppressive microenvironment (50); 2) In HCC, DCs express relatively higher levels of interleukin-10, prostaglandin E2, and indoleamine 2,3-dioxygenase as well as lower activity markers of an antigen-presenting cell, thereby reducing the activation of T-cell and giving rise to a specific ineffective T-cell population (51) (3); In the microenvironment of HCC, there exists an extensive population of immunosuppressive cells, such as Tregs and MDSCs (52, 53). Tregs inhibit T cell proliferation not only through releasing inhibitory cytokines (transforming growth factor β, interleukin-10, and interleukin-35) but also contacting the PD-L1/PD-1 pathway (54). In HCC, different inflammatory cytokines cooperatively disrupt the maturation of myeloid cells and drive the expansion of MDSCs. MDSCs restrain the anti-tumor response by upregulating PD-L1 expression, depleting essential amino acids, and expressing immunosuppressive cytokines (55); and (4) The tumor microenvironment of HCC is usually characterized by the presence of dysfunctional tumor-infiltrating lymphocytes (56). Depletion, senescence, and anergy state of T cells are all directly related to a failure of immunotherapy (57).

Hopefully, combination immunotherapies have been extensively explored to overcome immunotherapy resistance and improve the anti-tumor effect. Chemotherapy and radiation therapy, as the conventional genotoxic therapies, can induce an immunogenic form of cancer cell death (ICD) (58). ICD is characterized by the release of numerous damage-associated molecular patterns including calreticulin, ATP, and high mobility group protein B1. These molecules are liberated from dying tumor cells and recognized by specific pattern recognition receptors expressed by DCs, resulting in the recruitment of DCs, processing and presentation of tumor antigens, and activation of the anti-tumor immune response (59–61). In addition, chemotherapy can deplete certain immunosuppressive cells in the TME (such as MDSCs and Tregs) and benefit from the differentiation of tumor-associated macrophages with an anti-tumor phenotype. Adhesion molecules can be upregulated by radiation therapy to promote the recruitment of anti-tumor T lymphocytes to the tumor site (59, 62, 63). Therefore, chemotherapy and radiation therapy possess immunogenic effects as well as the ability to modulate the immune-suppressive microenvironment (64). These may be possible synergistic mechanisms underlying the effectiveness of combination immunotherapies for MPMs.

However, it is clear that not all ICI combination therapies could enhance immune activity. There are many existing problems, such as biomarker selection, the timing of administration, the optimal combination and sequence, and drug toxicities (65). Deeper research into synergistic mechanisms is critical for the design of efficient therapeutic schemes.

## Concluding Remarks

In conclusion, definite pictures of malignancy and the exclusion of metastatic tumors are important in the diagnosis of MPMs. As immune escape is a common pathological mechanism of various malignant tumors, ICI combination therapy may be a promising treatment strategy for MPMs. To our knowledge, this is the first case report of an MPM patient with PSC and HCC who responded to camrelizumab combination therapy. After disease progression, the patient also benefits from camrelizumab, which provides a promising option for MPMs. However, to maximize the clinical benefits of ICI therapy, it is essential to clarify the mechanism underlying the resistance.

## Data Availability Statement

The raw data supporting the conclusions of this article will be made available by the authors without undue reservation.

## Ethics Statement

Written informed consent was obtained from the individual(s) and minor(s)’ legal guardian/next of kin for the publication of any potentially identifiable images or data included in this article.

## Author Contributions

FL conceived and designed the idea, organized the case report overall, made critical appraisal, and approved the final manuscript. ZW and NY contributed to collecting and analyzing radiology figures. YW summarized the data and drafted the initial manuscript.

## Conflict of Interest

The authors declare that the research was conducted in the absence of any commercial or financial relationships that could be construed as a potential conflict of interest.

## Publisher’s Note

All claims expressed in this article are solely those of the authors and do not necessarily represent those of their affiliated organizations, or those of the publisher, the editors and the reviewers. Any product that may be evaluated in this article, or claim that may be made by its manufacturer, is not guaranteed or endorsed by the publisher.

## Abbreviations

MPMs, multiple primary malignancies; PSC, pulmonary sarcomatoid carcinoma; HCC, hepatocellular carcinoma; PD, progressive disease; PR, partial response; SD, stable disease; CTLA-4, cytotoxic T lymphocyte-associated protein 4; PD-1, programmed cell death protein 1; PD-L1, programmed death ligand 1; TKI, tyrosine kinase inhibitor; NMPA, National Medical Products Administration; FDA, Food and Drug Administration; MRI, magnetic resonance imaging; CT, computed tomography; TACE, transcatheter arterial chemoembolization; HBV, hepatitis B virus; HBsAg, hepatitis B surface antigen; HBeAg, hepatitis B e antigen; HBcAb, hepatitis B core antibody; AFP, alpha-fetoprotein; NGS, next-generation sequencing; TPS, tumor proportional score; DCs, dendritic cells; Tregs, regulatory T-cells; TIL, tumor-infiltrating lymphocyte; MDSCs, myeloid-derived suppressor cells; IDO, indoleamine 2,3-dioxygenase; APC, antigen-presenting cell; ICD, immunogenic cell death.
